# Editorial: Hepatic glucose and lipid metabolism

**DOI:** 10.3389/fphys.2022.1009566

**Published:** 2022-09-15

**Authors:** Huabing Zhang, Guang Ren, Aijun Qiao

**Affiliations:** ^1^ Department of Biochemistry and Molecular Biology, Metabolic Disease Research Center, School of Basic Medicine, Hefei, China; ^2^ The Affiliated Chuzhou Hospital of Anhui Medical University (The First People’s Hospital of Chuzhou), Chuzhou, China; ^3^ Division of Endocrinology, Diabetes, and Metabolism, Department of Medicine, University of Alabama at Birmingham, Birmingham, AL, United States; ^4^ Department of Biomedical Engineering, School of Medicine and School of Engineering, University of Alabama at Birmingham, Birmingham, AL, United States

**Keywords:** liver, glucose, lipid, metabolism, molecular mechanisms, type 2 diabetes, non-alcoholic fatty liver disease, crosstalk

In general, hepatic glycolipid metabolism is tightly regulated in healthy individuals. However, disorders of its metabolism can lead to several metabolic diseases, including diabetes, insulin resistance, obesity, non-alcoholic fatty liver disease (NAFLD), and atherosclerotic cardiovascular diseases, which are all rising rapidly worldwide. Thus, understanding the underlying mechanisms regulating hepatic glycolipid metabolism can provide potential targets and rationale for novel therapeutic strategies. This Research Topic mainly focuses on the molecular mechanisms by which novel regulators/pathways and crosstalk between the liver and other organs contribute to hepatic glycolipid metabolism.

## Signaling pathways regulating hepatic glycolipid metabolism

The liver is a central organ that precisely controls glucose and lipid homeostasis by various signaling and cellular pathways. Skewing regulation of these signaling pathways can cause dysfunction of hepatic glycolipid metabolism, thereby contributing to the development of metabolic diseases. Thus, understanding the molecular mechanisms underlying signaling pathways regulating hepatic glycolipid metabolism is vital to developing targeted therapies for metabolic diseases.

The serine/threonine kinase Akt, a key factor participating in multiple signaling pathways, has been shown to play a crucial role in hepatic glycolipid metabolism and is a core focus of current research on metabolic diseases. In this Research Topic of the journal, Miao et al. systemically reviewed the recent advances in Akt pathway-related metabolic diseases and medications and summarized the challenges and perspectives of Akt and its associated pathways as a potential drug target for the treatment of metabolic diseases.

ATP not only acts as an essential extracellular but also an intracellular molecule to modulate islet β cell functions and hepatic glycolipid metabolism. Impaired ATP metabolism is one of the critical events in the pathogenesis of pancreatic β cell and liver dysfunctions. In this Research Topic of the journal, Li et al. provided a comprehensive review of vital roles and mechanisms of intracellular and extracellular ATP signaling in regulating islet β cell functions and hepatic glycolipid metabolism and evidence of impaired ATP signaling associated with the pathogenesis of metabolic disorders, primarily type 2 diabetes (T2DM).

Empagliflozin, a sodium-dependent glucose transporter 2 (SGLT2) inhibitor, is first-in-class and an effective drug for the treatment of hyperglycemia in T2DM by reducing renal glucose reabsorption. However, whether the anti-diabetic effect of empagliflozin also relies on other mechanisms remains elusive. In this Research Topic of the journal, Yu et al. identified a previously unrecognized mechanism that empagliflozin lower blood glucose levels partially by suppressing hepatic gluconeogenesis and promoting glycogen synthesis via AMPK/CREB/GSK3b signaling pathway. Importantly, they observed that db/db mice treated with Empagliflozin can lead to a significantly reduced blood glucose level and body weight and improved glucose tolerance.

### Cellular and molecular mechanisms of NAFLD

NAFLD is a condition characterized by the presence of fat accumulation in more than 5% of hepatocytes and is the most common chronic liver disease in western countries. It is often associated with obesity, dyslipidemia, insulin resistance, and T2DM and is recognized as the hepatic manifestation of those diseases. The pathogenesis of NAFLD is highly complex and strongly associated with perturbations in hepatic lipid and glucose metabolism. By far, the molecular mechanisms underlying the development of NAFLD remain to be fully understood.

Increasing evidence has revealed that many adverse metabolic conditions have been linked with obstructive sleep apnea (OSA), including insulin resistance and T2DM. However, the relationship between OSA and NAFLD needs further study. In this Research Topic of the journal, Wang et al. reported that chronic intermittent hypoxia (CIH) is a pathological characteristic of OSA and is associated with the pathogenesis of NAFLD. Mechanistically, they revealed that persistent CIH could result in dysregulated hepatic autophagic activity, steatosis, and ER stress, highlighting CIH as a potential therapeutic target for intervention and prevention of OSA-associated NAFLD.

The impairment of lipid processing pathways, particularly the condensation of β-oxidation-derived acetyl-CoA into the ketogenic pathway, is closely associated with the deregulation of hepatic glycolipid metabolism. Moreover, the administration of ketone bodies for mice and human patients demonstrates a significant improvement in NAFLD. In this Research Topic of the journal, Mooli et al. summarized the pathway and regulation of hepatic ketone body production. They further reviewed the role of ketogenesis in the occurrence of NAFLD and nonalcoholic hepatitis and the underlying molecular mechanisms. Moreover, they prospected the possibility of a ketogenic diet in the treatment of NAFLD.

### Crosstalk between liver and other metabolic tissues

It is well known that the liver also controls systemic metabolism as a paracrine and endocrine communication regulator. This special edition emphasizes the “crosstalk,” a scenario that may potentially be implemented into clinical practice.

In this Research Topic of the journal, Rodriguez-Calvo et al.collected data from 389 individuals with diabetes and metabolic syndrome to investigate the relationship between circulating fatty acid binding protein 4 (FABP4) levels and liver steatosis. They found that circulating FABP4 level is upregulated in patients with metabolic diseases and strongly associated with fatty liver index (FLI) in both linear and logistic regression analyses. This study also proposes serum FABP4 as a molecular biomarker of the early stages of NAFLD in individuals at increased cardiometabolic risk. The circulating FABP4 associated with FLI could potentially indicate cross-tissue signals in intracellular lipid metabolism and provide clinical relevance to cardiovascular diseases.

Further insights into diabetic cardiomyopathy (DCM) were provided by Hao et al. The authors utilized a UPLC-MS/MS approach to analyze metabolites from the plasma of 78 total patients with diabetes (39 diabetes with DCM and 39 diabetes without DCM as controls). The authors analyzed a total of 2,806 biochemicals. In KEGG enrichment analysis, they identified three signaling pathways that are significantly altered in DCM pathogenesis: porphyrin metabolism, chlorophyll metabolism, and lysine degradation. Notably, the evidence that: the accumulation of porphyrin increases the risks of cirrhosis and liver cancer, chlorophyll can reduce glucose-induced oxidative stress and decreases the incidence of liver tumors, and Lysine level is strongly associated with the degree of hepatic insulin resistance. Therefore, this study enhances the understanding of DCM’s pathogenesis, occurrence, and development. It also builds a bridge that links DCM metabolites to potential therapeutic targets for liver diseases.

## Summary

The articles published in this Research Topic provide the most recent advancements in novel factors/signaling pathways regulating hepatic glycolipid metabolism, the new pathogenic mechanisms of NAFLD, and the crosstalk between the liver and other metabolic tissues linked with the development of metabolic diseases. The Editors are pleased to highlight their work and hope that it may provide a foundation for future studies that could lead to the development of potential therapeutic strategies for metabolic diseases including NAFLD, obesity, and T2DM.

